# Drug repurposing of nitazoxanide: can it be an effective therapy for COVID-19?

**DOI:** 10.1186/s43141-020-00055-5

**Published:** 2020-07-28

**Authors:** Dina B. Mahmoud, Zayyanu Shitu, Ahmed Mostafa

**Affiliations:** 1 Pharmaceutics Department, National Organization for Drug Control and Research, Giza, Egypt; 2 Hospital Services, Management Board, Ministry of Health, Zamfara State, Gusau, Nigeria; 3 Centre of Scientific Excellence for Influenza Viruses, National Research Centre, Cairo, Egypt

**Keywords:** Broad spectrum antiviral, Bronchodilation, COVID-19, Cytokines, Repurposed nitazoxanide

## Abstract

**Background:**

The current outbreak of pandemic coronavirus disease 2019 (COVID-19) aggravates serious need for effective therapeutics. Over recent years, drug repurposing has been accomplished as an important opportunity in drug development as it shortens the time consumed for development, besides sparing the cost and the efforts exerted in the research and development process.

**Main body of the abstract:**

The FDA-approved antiparasitic drug, nitazoxanide (NTZ), has been found to have antiviral activity against different viral infections such as coronaviruses, influenza, hepatitis C virus (HCV), hepatitis B virus (HBV), and other viruses signifying its potential as a broad spectrum antiviral drug. Moreover, it has been recently reported that NTZ exhibited *in vitro* inhibition of SARS-CoV-2 at a small micromolar concentration. Additionally, NTZ suppresses the production of cytokines emphasizing its potential to manage COVID-19-induced cytokine storm. Furthermore, the reported efficacy of NTZ to bronchodilate the extremely contracted airways can be beneficial in alleviating COVID-19-associated symptoms.

**Short conclusion:**

All these findings, along with the high safety record of the drug, have gained our interest to urge conductance of clinical trials to assess the potential benefits of using it in COVID-19 patients. Thus, in this summarized article, we review the antiviral activities of NTZ and highlight its promising therapeutic actions that make the drug worth clinical trials.

## Background

**Fig. 1:**
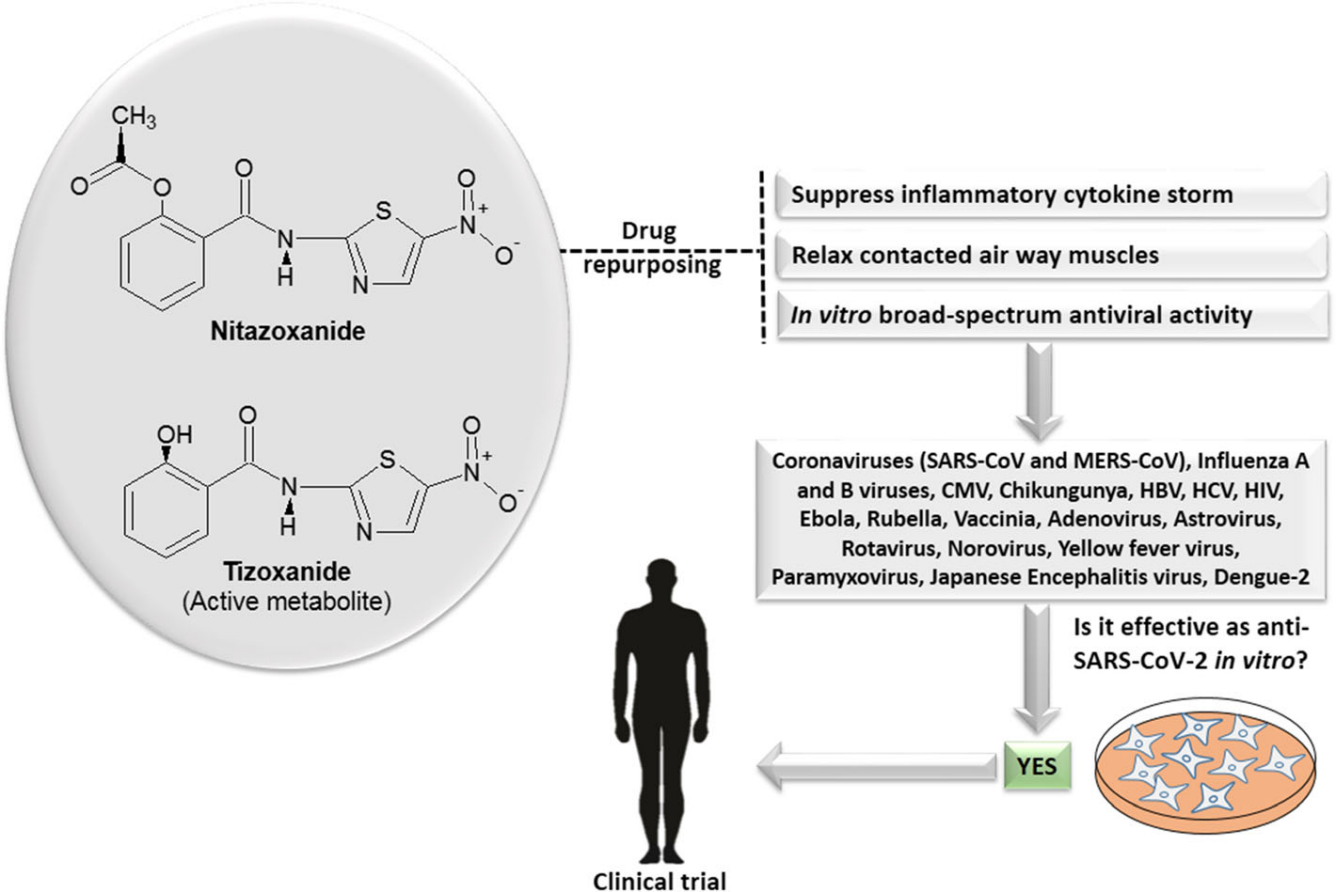
Drug repurposing of nitazoxanide (NTZ) and its active metabolite tizoxanide (TIZ). NTZ represents a promising medication for clinical trial against COVID-19 due to its ability to control excessive inflammatory immune responses, its bronchodilator effect, and *in vitro* anti-SARS-CoV-2 activity

The outbreak of recent coronavirus disease 2019 (COVID-19) was firstly identified in Wuhan, China [1], by the end of the year 2019. The main cause for this disease was a novel beta-coronavirus that was recognized as severe acute respiratory syndrome coronavirus-2 (SARS-CoV-2) by the International Committee on Taxonomy of Viruses [2]. Coronaviruses belong to Coronaviridae family within the order Nidovirales. They are enveloped and positive-sense single-stranded RNA viruses. Several coronaviruses infect humans and other mammalian hosts. Coronaviruses are classified into four distinct genera (alpha, beta, delta, and gamma), and among them, few alpha- and beta-coronaviruses are recognized to infect humans [3]. Coronaviruses are usually zoonotic; however, efficient human-to-human transmission was observed with SARS-CoV-2 rather than SARS-CoV and Middle East respiratory syndrome coronavirus (MERS-CoV) [4]. By June 30, 2020, the World Health Organization (WHO) recorded more than 10 million cases of COVID-19 with nearly half a million deaths worldwide [5], demonstrating that COVID-19 is a critical public health threating disease. Currently, there is neither clinically approved nor currently available antiviral drug for coronavirus treatment. Though remdesivir advanced into human clinical trials as an antiviral agent to treat COVID-19, it is still critically and urgently needed to explore other effective drugs as potential therapy for COVID-19 [6]. Over recent years, drug repurposing has been accomplished as an important opportunity in drug development and as a smart strategy involves recognizing new indications for the currently approved or investigated compounds that are out of the scope of their usual medical indications. This strategy is of great interest as it shortens the time consumed for development and safety assessment, besides sparing the cost and the efforts exerted in the research and development process. Moreover, it offers the advantage of decreasing the possibility of drug failure especially from the safety considerations as most of the drugs are repurposed after proving their safety in preclinical or clinical trials [7]. Nitazoxanide (NTZ) was approved in the USA as an antiparasitic therapy for diarrhea and enteritis triggered by *Cryptosporidium* spp. and *Giardia lamblia* in 2002 [8]. It targets host pathways by the disruption of mitochondrial respiration [9]. Regarding the safety of NTZ, it was reported that above 75 million patients (including children) administered the drug in post-marketing experience for the treatment of parasitic intestinal infections with no observed critical safety issues correlated to the drug. Besides the antiparasitic action of NTZ, *in vitro* assessment of the drug revealed an inhibitory action against a wide range of anaerobic gram-positive and gram-negative bacteria along with replicating and non-replicating strains of *Mycobacterium tuberculosis* [10]. Moreover, the activity of NTZ as a treatment for *Clostridium difficile* disease has been confirmed by animal studies as well as clinical trials [9]. Serendipitous suspicion of the antiviral effect of NTZ was raised while treating cryptosporidial diarrhea in patients suffering co-infection of acquired immune deficiency syndrome (AIDS), with hepatitis B virus (HBV) or hepatitis C virus (HCV) [11]. Since then, extensive investigation of the antiviral therapeutic potential of NTZ and its active metabolite tizoxanide (TIZ) has been conducted. Our aim in this article is to enumerate the exceptional antiviral effects of NTZ and highlight its therapeutic potential to combat the novel COVID-19 (Fig. 1).

## Main text

### Nitazoxanide and viral infections

Nitazoxanide (NTZ) showed a promising activity against a broad spectrum of viruses as summarized in Table 1.

**Table 1:** The inhibitory effect of NTZ and tizoxanide (TIZ) as broad spectrum antivirals

Virus	Dose	Compound	Ref
Canine coronavirus S-378	IC_50_, 1 μg/ml	TIZ	[9]
Bovine coronavirus (L9)	IC_50_, 0.3 μg/ml	NTZ	[12]
Murine coronavirus	IC_50_, 0.3 μg/ml	NTZ	[12]
Mouse hepatitis virus (A59)	IC_50_, 0.3 μg/ml	NTZ	[12]
Human enteric coronavirus (4408)	IC_50_, 0.3 μg/ml	NTZ	[12]
MERS-CoV	IC_50_, 0.92 μg/ml	NTZ	[12]
MERS-CoV	IC_50_, 0.83 μg/ml	TIZ	[12]
SARS-CoV-2	IC_50_, 2.12 μM	NTZ	[1]
Influenza A strains	IC_50_, 0.2–1.5 μg/ml	TIZ	[9]
Influenza B/Parma/3/04	IC_50_, 0.2–1.5 μg/ml	TIZ	[9]
Rotavirus: the human Wa-G1P (8)	IC_50_, 1 μg/ml	TIZ	[9]
Rotavirus: simian SA11-G3P (2)	IC_50_, 0.5 μg/ml	TIZ	[9]
Norovirus G1 replicon assay system	IC_50_, 0.5 μg/ml	TIZ	[13]
Human astrovirus	IC_50_, 1.47 μM	NTZ	[14]
Adenovirus enteritis	500 mg twice daily	NTZ	[15]
Paramyxovirus	IC_50_, 10 μg/ml	NTZ	[16]
Ebola virus	IC_100_, 20–40 μM	NTZ	[17]
HCV genotype 1a	IC_50_, 0.09 μg/ml	TIZ	[18]
HCV genotype 1b	IC_50_, 0.06 μg/ml	TIZ	[18]
HBV in clinical trials	500 mg daily	NTZ	[11]
Chikungunya virus	IC_50_, 2.96 μM	NTZ	[19]
Rubella virus	IC_50_, 0.35 μg/ml	NTZ	[20]
Vaccinia virus	IC_50_, 2 μM	NTZ	[21]
HIV	IC_50_, 0.5 μg/ml	NTZ	[22]
HIV	IC_50_, 0.5 μg/ml	TIZ	[22]
Human cytomegalovirus	IC_50_, 3.2 μM	NTZ	[23]
Dengue-2 virus	IC_50_, 0.1 μg/ml	TIZ	[24]
Yellow fever virus	IC_50_, 0.06 μg/ml	TIZ	[24]
Japanese encephalitis	IC_50_, 0.12 μg/ml	NTZ	[25]

#### Nitazoxanide and coronaviruses

Coronaviruses are a number of enveloped, non-segmented positive-sense RNA viruses characterized by a genome size which is very large as it ranges from about 27 to 34 kb. The respiratory infections caused by human strains, namely HCoV-229E, HCoV-OC43, HCoV-NL63, and HCoV-HKU1, are typically mild and self-limited, for instance common cold. On the other hand, epidemic and pandemic serious diseases are caused when humans are infected with three highly pathogenic beta-coronaviruses namely SARS-CoV, MERS-CoV, and the latest SARS-CoV-2 [6]. Currently, there is an urgent demand for new drug discovery to manage the infections caused by coronaviruses due to the lack of effective treatment options.

*In vitro* studies revealed that tizoxanide (TIZ) could inhibit the *in vitro* canine coronavirus S-378 replication in the infected A72 cells (the reported half maximum inhibitory concentration, IC_50_, is 1 μg/ml [9]). Cao et al. reported that NTZ could effectively inhibit bovine coronavirus (L9), murine coronavirus, mouse hepatitis virus (A59), and human enteric coronavirus (4408) cultured in mouse astrocytoma (DBT) and fibroblast (17Cl-1) cell lines in an IC_50_ of about 0.3 μg/ml; this effect involved inhibition of the expression of the viral N protein [12]. It is notable that both NTZ and its active metabolite (TIZ) generally depict comparable *in vitro* inhibition action on viruses; they inhibit MERS-CoV grown in LLC-MK2 cell lines (the reported IC_50_ = 0.92 and 0.83 μg/ml, respectively).

In 2020, after the breakdown of COVID-19, Wang et al. have reported that NTZ could inhibit SARS-CoV-2 in Vero E6 cells (ATCC-1586) after infection with SARS-CoV-2/Wuhan/WIV04/20192 at a low micromolar concentration (a half maximum inhibition concentration of 2.12 μM) [1]. Recently, Kelleni has suggested using NTZ with azithromycin as a protocol for early cases of COVID-19 [26]. Additionally, Pepperrell et al. reviewed the reported clinical research studies about NTZ to determine the safety of the drug and they calculated the minimum cost of drug production for the expected potential use in the treatment of COVID-19 [27]. Currently, there are 14 clinical trials for using NTZ either alone or in combination with other drugs (ivermectin or hydroxychloroquine) to treat COVID-19 patients; among them, 7 studies started recruiting patients as shown in Table 2. The other clinical trials are not recruiting patients yet. The countries that started clinical trials for NTZ are Egypt, the USA, Brazil, and Mexico [28].

**Table 2:** Current clinical trials to assess NTZ for treatment of COVID-19

COVID-19-related clinical trials	Country	Status
NTZ with ivermectin vs ivermectin with chloroquine	Egypt	Recruiting
NTZ for moderate case hospitalized patients	Brazil	Recruiting
NTZ for post-exposure prophylaxis in healthcare workers vs placebo vs dietary supplement	USA	Recruiting
NTZ 500 mg oral tablet	Mexico	Recruiting
NTZ vs hydroxychloroquine	Mexico	Recruiting
NTZ vs favipiravir vs chloroquine	Egypt	Recruiting
NTZ vs placebo	Egypt	Recruiting

#### Nitazoxanide and influenza viruses

NTZ readily undergoes hydrolysis and converts to TIZ in aqueous buffers as a cell culture medium [28, 29]. Several *in vitro* studies revealed that TIZ caused inhibition of the replication of sixteen various strains of influenza A viruses (IAV) [A/Puerto Rico/8/34 (H1N1, A/H1N1-PR8), A/Wisconsin/33 (H1N1), A/Parma/24/09 (H1N1, oseltamivir-resistant), A/Goose/Italy/29624603 (H1N1), A/California/04/p2009 (H1N1), A/Ohio/88/2012 (H3N2v), A/Ohio/83/2012 (H3N2v), A/Washington/01/2007 (H3N2), A/Texas/12/2007 (H3N2), A/Firenze/7/03 (H3N2), A/Parma/6/07 (H3N2, amantadine-resistant), A/Canine/Colorado-1/224986/06 (H3N8), A/Canine/Colorado-3/3/06 (H3N8), A/Canine/Colorado-4/2025974/07 (H3N8), A/Chicken/Italy/9097/97 (H5N9, A/H5N9), and A/Turkey/Italy/RA5563/99 (H7N1)] and one influenza B virus (IBV) [Influenza B/Parma/3/04]. The half maximal inhibitory concentration of TIZ against influenza viruses ranged from 0.2 to 1.5 μg/ml [9]. It was also reported that IAV is not likely to develop resistance to TIZ since thiazolides do not have a direct action against viral RNA or protein [30], and it is reported that the development of viral resistance to the antiviral drugs that target host cell is less expected when compared to virus-specific inhibitors [31]. Moreover, a synergistic antiviral effect was observed upon combining NTZ with oseltamivir or zanamivir against influenza A/H1N1-PR8 and the avian A/H5N9 [32].

Tilmanis et al. have reported the potent antiviral effect of TIZ against 210 seasonal influenza viruses as well as viruses that exhibit resistance to neuraminidase inhibitors including oseltamivir, zanamivir, peramivir, and laninamivir [33].

In a human clinical trial involving 257 patients, NTZ could effectively reduce the duration of symptoms of influenza in patients that received oral doses of 600 mg twice daily comparing to the placebo. Additionally, there was a significant decrease in the viral titers in this group of patients when compared to patients that received placebo (*p* = 0.0006). Interestingly, no observable antiviral resistance against NTZ was reported among treated influenza viruses and no adverse events were reported on humoral immune response in the drug-receiving patients [34].

#### Nitazoxanide and rotavirus

Viral gastroenteritis that affect children are mostly self-limited infections, and the use of antimicrobial drugs may be not required; however, the use of antimicrobials may be reasonable as diarrheal illness is responsible for more than one million child mortalities on an annual basis in the developing countries [35]. In the developing countries, gastroenteritis disease in pediatric population is mostly caused by rotavirus. Furthermore, it may lead to 500,000 deaths annually in children under the age of 5 years [36]. TIZ was reported to hinder the replication of two strains of rotavirus which are the human Wa-G1P (8) and simian SA11-G3P (2) at an IC_50_ of 1 and 0.5 μg/ml, respectively [9].

Few clinical trials have revealed the potential benefits of using NTZ against rotavirus causing gastroenteritis. The benefits include reduction in the duration of rotavirus disease in hospitalized pediatric population as compared to placebo, thus signifying that NTZ is a vital treatment option for diarrhea caused by rotavirus [37, 38].

#### Nitazoxanide and norovirus

Norovirus is progressively recognized as a major cause of viral gastroenteritis in immunocompromised patients [39]. In 2011, the assessed number of childhood deaths worldwide as a result of norovirus infections is 71,000 [40]. The half maximal inhibitory concentration of TIZ was 0.5 μg/ml, in a norovirus G1 replicon assay system utilizing HG-23 cells. Additionally, NTZ was reported to treat norovirus gastroenteritis in a 43-year-old case who was diagnosed with leukemia and subjected to chemotherapy in addition to transplantation of hematopoietic stem cell; the patient experienced an improvement after 24 h post-treatment [13].

Results of a randomized, 2-phase, double-blind clinical study which was conducted enrolling 200 subjects (including 50 adults and 150 children from 2 months to 11 years old) with viral gastroenteritis induced by the infection with norovirus or rotavirus signified the ability of NTZ for the significant reduction in the duration of disease symptoms when compared with the placebo (*p* = 0.0295) [41]. Morris and Morris have reported an improvement of diarrhea, nausea, and abdominal pain after 2 days of NTZ therapy in a clinical study enrolling 14 patients diagnosed with viral gastroenteritis because of norovirus [42].

Dang et al. have reported a synergistic inhibitory effect of human norovirus replication and complete depletion of the replicons from the host cells after administration of combination therapy of ribavirin and TIZ. Briefly, their results revealed that TIZ inhibits viral replication by stimulation of cellular antiviral response particularly through expression of interferon regulatory factor 1. Treatment with NTZ alone or in combination with ribavirin represents a promising strategy for combating norovirus-induced gastroenteritis principally in immunosuppressed patients [14].

#### Nitazoxanide and astrovirus

Human astrovirus is known to cause 2 to 9% of acute non-bacterial diarrhea pediatric cases globally. Human astrovirus infections can be fatal particularly in immunocompromised patients as the infection may be associated with necrotizing enterocolitis and severe diarrhea, in addition to other fatal diseases such as respiratory diseases [43], encephalitis, and meningitis [44]. In spite of the high prevalence of astrovirus and the risk of the associated severe diseases, there is currently no vaccine or drug therapy that is available for combating this virus. Hargest et al. have recently reported the first evidence that NTZ can cause *in vitro* inhibition of the replication of several astrovirus strains as well as in vivo reduction in the viral shed and diarrhea in symptomatic astrovirus-infected turkey poultry model [43]. Their results revealed that NTZ was superior to ribavirin, foscarnet, and acyclovir as they failed in the inhibition of human astrovirus replication even at elevated concentrations (250 μM). On the other hand, NTZ could effectively inhibit viral replication in a manner proportional to concentration with an IC_50_ of 1.47 μM.

#### Nitazoxanide and adenovirus

Human adenoviruses are non-enveloped, double-stranded DNA viruses with icosahedral capsids. Infection with human adenovirus usually leads to severe life-threatening disease particularly in immunocompromised subjects. At the moment, there is a lack of available specific antiviral drugs against adenovirus infections [6]. Garrigos et al. reported the clinical efficacy of NTZ against adenovirus-related enteritis in a 61-year-old immunocompromised hospitalized patient; the authors detected a speedy recovery of diarrhea and viremia along with a negative PCR result 2 weeks post-initiation of NTZ therapy of 500 mg two times daily dosing regimen. Interestingly, the patient recovered from diarrhea after 48 h of treatment. It is notable that the limited toxicity and viral resistance of NTZ, as well as the few drug interactions, make it a promising treatment option for such infections [15].

#### Nitazoxanide and paramyxovirus

Paramyxoviruses are a group of airborne viruses which are responsible for some respiratory diseases. They include agents of measles, mumps, respiratory syncytial virus, Newcastle disease in poultry, and parainfluenza in human. These viruses are characterized by single-stranded RNA genome and polymerase. They have an envelope of a diameter between 150 and 300 nm. The viruses had been reported in some species of snakes which is transmitted to human [45]. They are highly contagious viruses which induce infection associated with respiratory signs in human, and they are transmitted by respiratory secretions [46].

Piacentini et al. identified in their study the anti-paramyxovirus effect of NTZ; they found that NTZ halts the F-trafficking to the plasma by acting on Sendai virus and RSV F-protein aggregate of the viral cell. They further found NTZ acting as an ERp57 non-competitive blocker; the NTZ binding site is said to be located at the ERp57-b/b′ non-catalytic domain border [16].

#### Nitazoxanide and Ebola virus

Ebola virus is an enveloped negative single-stranded RNA virus. Ebola virus is transmitted to human through wild animals, and it can further spread between humans via transmission from subject to subject. Ebola virus infection is identified as Ebola hemorrhagic fever; it exhibited high mortality rates (25 to 90% in the previous outbreaks) [6]. Jasenosky and his colleagues have recently reported that NTZ could effectively amplify the host innate immune response and inhibit the replication of Ebola virus. The reported mode of action involves enhancing retinoic-acid-inducible protein I-like receptor as well as mitochondrial antiviral signaling protein. Moreover, NTZ stimulates the expression of interferon regulatory factor 3 and prompts antiviral phosphatase GADD34 transcription thus presenting a promising approach as a therapy for Ebola virus prone disease [17].

#### Nitazoxanide and hepatitis C virus

HCV is an enveloped positive-sense single-stranded RNA virus belonging to the family Flaviviridae. The virus can be transmitted essentially through blood to cause either acute or chronic hepatitis. HCV is encountered as the essential cause of hepatic cancer [6]. TIZ activity against HCV was primarily investigated in genotype 1a in the HCV replicon cell culture system grown in AVA5 cells, and genotype 1b in the HCV replicon replicated in Huh7.5 cell lines. The TIZ IC_50_ for genotype 1a was 0.09 μg/ml, while for genotype 1b, it was 0.06 μg/ml. Synergism was also reported upon combining TIZ with interferon α or with 2′C methylcytidine [18]. It is noteworthy to mention that HCV did not show decreased sensitivity to TIZ. Moreover, the sequencing of the viral genome did not experience mutations to develop resistance. Thus, a host-targeted mechanism of action was suggested [47].

Clinical trials were conducted to assess the efficacy of NTZ against HCV with promising outcomes as patients accomplished a sustained virological response without traceable viral RNA in serum after NTZ therapy [11, 48]. Even though the improved outcomes were achieved upon the administration of NTZ combined with peginterferon (PegIFN) as a dual therapy for chronic hepatitis C, development was ceased as a result of discovery of novel direct acting antiviral compounds. Assessment of combinations of NTZ with antiviral agents acting directly for combating chronic HCV in patients co-infected with HIV may be an interesting research area for future advance.

#### Nitazoxanide and hepatitis B virus

HBV is a source of significant progressive liver disease; an estimated number of two billion people are living with HBV in the world, out of which approximately 360 million people are infected chronically [49]. Korba et al. showed how NTZ and its metabolite, TIZ, inhibit HBV DNA and its core antigen. The antigens are of two types, the HBeAg and HBsAg, and they were inhibited in prepared cell cultures. Both molecules were found to be more effective than other antiviral drugs such as lamivudine (LMV) and adefovir dipivoxil (ADV), as they exerted activity against six HBV mutants that were resistant to LMV and ADV [18]. In a recent study, Sekiba and others investigated the inhibitory effect of NTZ against not only the DNA but also the RNA of the HBV virus. They have shown that NTZ targets the protein HBx-damage specific DNA-binding protein 1 (DDB1) interaction thereby inhibiting the expression of HBV cccDNA and RNA transcription [50]. A recent *in vitro* study showed that NTZ acts by leading HBsAg loss by restraining serum HBV DNA. NTZ shows an original mechanism of antivirals with the inhibition of oxidative phosphorylation in the mitochondria [51]. Furthermore, clinical trial using 500 mg dose of NTZ given daily for 12 consecutive months to 12 adults patients with hepatitis B showed its effect on HBV. The findings showed that 4 patients of the 12 exhibited HBeAg-positive result and 8 patients exhibited negative HBeAg result. These occurred in an average of 3 months. It clearly shows the therapeutic potential of NTZ on HBV patients [11].

#### Nitazoxanide and chikungunya virus

Chikungunya virus is a positive-sense single-stranded RNA virus. Chikungunya virus causes chikungunya disease that is identified by fever, conjunctivitis, arthritis, and severe arthralgia. The infection is transmitted by the female mosquitoes infected with the virus. In 2005, the outbreak of the disease occurred in France, and then, the infection spread to many other countries with hundreds of deaths and greater than one million infected cases [52]. Chikungunya virus is identified as a risk group-3 pathogen [53]. This group of pathogens can enter the cells via receptor-dependent endocytosis. During the entry, conformational changes occur in the viral envelope glycoproteins E1 and E2 [54] which lead to the formation of trimers with subsequent fusion between virus and endosome membranes [55] and release of the viral genome into cytosol. At present, there is no effective approved antiviral drug for treatment of chikungunya infection [6]. A recent study has shown that NTZ could limit both the entry and the release of the virus as well as cell-to-cell transmission. Furthermore, the drug exhibited broad antiviral activity against two clinical chikungunya virus isolates in addition to two types of alphaviruses namely Sindbis virus and Semliki forest virus [19].

#### Nitazoxanide and rubella virus

Rubella virus is characterized as a single-stranded positive RNA genome. It is small and enveloped [56]. The disease that is induced by rubella virus is contagious, characterized by rashes, and it is preventable by vaccines. It causes persistent fetus infection during pregnancy, leading to birth defects. The NTZ exerts its significant effect on rubella virus by inhibiting its replication in a primary culture of human umbilical vein endothelial cells in a concentration-dependent manner with an IC_50_ of 0.35 μg/ml [20].

#### Nitazoxanide and vaccinia virus

Vaccinia virus is utilized as a vector for the delivery of the other antigens [57]. Nevertheless, the virus is able to induce a severe disease in immunosuppressed subjects and also in patients suffering from eczema. Hickson et al. investigated the potential antiviral effect of NTZ against vaccinia virus. Their study demonstrated that NTZ could inhibit vaccinia virus replication with an IC_50_ of 2 μM [21].

#### Nitazoxanide and human immunodeficiency virus

HIV infection is devastating to humans since 30 years as a minimum. Since then, HIV infected 60 million subjects and caused more than 25 million deaths. Highly active antiretroviral therapy is available and effective treatment strategy for acquired immune deficiency patients; this strategy typically includes a combination of three drugs from two or more classes. For instance, two drugs belonging to the class of nucleoside reverse transcriptase inhibitors are combined with a third drug from these classes: integrase inhibitors, non-nucleoside reverse transcriptase inhibitors, or protease inhibitors. As a result of the high cost of the currently available drugs and the potential serious side effects, development of new antiviral drugs is needed [58]. Tan et al. found that both NTZ and the metabolite TIZ could effectively inhibit the replication of HIV (IC_50_ was about 0.5 μg/ml). Additionally, NTZ exhibited synergistic antiviral effects when combined with some HIV drugs including inhibitors of integrase (raltegravir), nucleoside, and non-nucleoside reverse transcription namely azidothymidine and efavirenz, respectively [22].

#### Nitazoxanide and human cytomegalovirus

More than 60% of human population is susceptible to human cytomegalovirus infections globally. The highly risk group of population includes acquired immune deficiency patients and immunocompromised subjects since the significances of cytomegalovirus infections may cause severe and life-threatening complications [59]. Mercorelli et al. have recently reported that NTZ specifically targets viral transcription factor IE2 functions thus blocking the replication of the virus with an IC_50_ of 3.2 μM. These findings signified a promising antiviral effect of NTZ that is needed to treat congenital infections and the development of strains that can resist the current anti-DNA polymerase compounds [23].

#### Nitazoxanide and other viruses

TIZ could effectively inhibit the replication of both dengue-2 and yellow fever in Vero cell lines with half maximal inhibitory concentrations of 0.1 and 0.06 μg/ml, respectively [24]. Another study depicted the activity of NTZ in the inhibition of Japanese encephalitis viral replication in BHK-21 cells with an IC_50_ of 0.12 μg/mL; in vivo activity of NTZ was also studied on mice infected with a fatal dose of Japanese encephalitis virus, and the results revealed that the infected mice could survive after they received NTZ orally for a period of 25 days. On the other hand, all untreated mice died on the ninth day of the experiment. The survival of the mice was proportional to the administered doses as the observed survival was 30%, 70%, and 90% for NTZ doses of 50, 75, and 100 mg/kg/day, respectively [25].

### Nitazoxanide and pro-inflammatory mediators

Besides the antiviral activity of NTZ, the ability of NTZ to inhibit pro-inflammatory cytokine production including TNFα, interleukin (IL)-2, IL-4, I-5, IL-6, IL-8, and IL-10 in PBMC (peripheral blood mononuclear cells) was also reported [60]. Other studies support these findings reporting that 100 mg/kg oral dose of NTZ when given to mice 2 h prior to 1 ml of 4% thioglycollate injected intraperitoneally decreased plasma IL-6 levels by 90% when compared to vehicle [61]. The findings suggest that NTZ can improve the cytokine storm that may be experienced with severe COVID-19 patients [62] through suppression of the overproduction of pro-inflammatory cytokines.

### Nitazoxanide and bronchodilatation

In a recent study, Miner and his colleagues reported that NTZ is a potent Ca^+2^-activated Cl^−^ channel (TMEM16A) antagonist that causes blockade in the contraction and depolarization of the airway smooth muscles. They assessed NTZ efficacy in harsh conditions utilizing extremely contracted airways or airways that were subjected to a cytokine cocktail, and the results revealed that the drug could surprisingly bronchodilate airways. Their findings suggest the potential benefit of using NTZ in COVID-19 patients as it may help to alleviate the disease-associated symptoms [63].

## Discussion and future perspectives

NTZ exhibits extraordinary broad spectrum antimicrobial activity [64]. In the USA, NTZ gained FDA approval as an antiparasitic drug for the treatment of cryptosporidiosis and giardiasis infections. Owing to its safety and activity against a wide range of viral infections, it is currently under assessment in many clinical trials for a number of infectious viral diseases [65, 66]. The standard treatment with 500 mg dose of NTZ adequately achieves TIZ plasma levels greater than 10 μM within 1 h post-dosing, and plasma levels of TIZ are maintained above that concentration until about 5 h after dosing with a maximum plasma concentration of ~ 35 μM [67]. After 10 h post-dosing, the plasma levels of TIZ start to drop below the IC_50_ signifying that the standard dosing regimen of NTZ will be adequate to maintain effective antiviral concentrations throughout the treatment. A published study reported that the administration of a high single dose (4 g) of NTZ achieved a maximum plasma concentration of approximately 200 μM in patients and even this high dose was well tolerated without toxicity [68]. These data suggest the possibility of combating COVID-19 as the reported IC_50_ against SARS-CoV-2 is only 2.12 μM, and that can be easily achieved with the typical dosing of NTZ.

The COVID-19 outbreak is considered as a threat for public health and a global emergency to search for novel effective treatment options to hinder the rapid spreading of the lethal COVID-19 which is urgently necessitated. Drug repurposing campaigns can reduce the cost and time required for developing new molecules. It is recently reported that some screened current drugs such as NTZ, chloroquine, and remdesivir were proved to inhibit the fatal SARS-CoV-2 using low concentrations in cultured Vero E6 cell lines with IC_50_ values of 2.12 μM, 1.13 μM, and 0.77 μM, respectively. It is reported in a Chinese study that oseltamivir, which is a drug that is commonly used for influenza A and B, had no positive effects as a treatment for COVID-19 [69]. However, oseltamivir is currently investigated as an antiviral drug in some clinical trials [70]. Additionally, ivermectin which possesses a potent anthelmintic effect has recently exhibited an *in vitro* inhibitory effect against SARS-CoV-2 within 48 h with an IC_50_ of approximately 2 μM [71]. There are some limitations of using these repurposed drugs; for example, the use of ivermectin may include some safety issues as the safety data on using the drug in children weighing less than 15 kg are inadequate [72]. Moreover, the use of ivermectin in pregnancy may be limited as it is categorized as a FDA class C drug and there is no sufficient data on its safe use during pregnancy [73]. Furthermore, chloroquine may induce toxicities when used for old patients or above dosing limits; several severe side effects can occur during fetus development if chloroquine is used during pregnancy. Additionally, high doses of chloroquine or hydroxychloroquine can exacerbate retinopathy [74]. Although the FDA has just granted an emergency approval for chloroquine and hydroxychloroquine use in COVID-19 [75], this authorization has recently been revoked based on the latest results of the clinical trials which revealed that these drugs exhibited no benefits in decreasing mortality or accelerating recovery and these results are in accordance with the recent data that revealed that these drugs are not likely to cause inhibition of SARS-CoV-2. Since COVID-19 is a pandemic disease, the cost of treatment is of great importance. The treatment options need to be accessible at affordable prices especially to middle- and low-income countries. Also in high-income countries, the burden of COVID-19 necessitates the availability of drugs at minimal costs [76]. Newly developed antiviral drugs such as remdesivir, favipiravir, and lopinavir/ritonavir are available at higher costs, and this should be taken into consideration during screening therapies available for SARS-CoV-2 infections. On the other hand, NTZ is one of the most promising treatment options for COVID-19 based on the post-marketing experience which demonstrated high safety record in both adult and children as aforementioned. Moreover, NTZ is classified as a pregnancy class B drug by the FDA [77]. Taking into consideration all these data, along with the broad spectrum antiviral properties of NTZ and its affordable price, suggests that NTZ can be a cost-effective and safe drug therapy against SARS-CoV-2. Consequently, we envision that repurposing NTZ as an effective antiviral therapy in fast-track clinical trials may combat the fatal COVID-19 and save the lives of a large number of patients.

## Conclusion

In conclusion, the available reported data suggest the potential therapeutic effects of NTZ in the treatment of SAR-CoV-2 with particular emphasis on (i) *in vitro* inhibitory action of NTZ against SARS-CoV-2 and other coronaviruses with easily attainable concentration, (ii) the inhibitory effect against pro-inflammatory cytokines including the suppression of IL-6 production, (iii) bronchodilatory effect, and (iv) a satisfactory safety record verified by the clinical trials and in extensive post-marketing experience.

## Data Availability

Not applicable

## Abbreviations

COVID-19: Coronavirus disease of 2019
SARS-CoV: Severe acute respiratory syndrome coronavirus
MERS-CoV: Middle East respiratory syndrome coronavirus
AIDS: Acquired immune deficiency syndrome
HBV: Hepatitis B virus
HCV: Hepatitis C virus
HIV: Human immunodeficiency virus
PBMC: Peripheral blood mononuclear cells
IL: Interleukin
NTZ: Nitazoxanide
TIZ: Tizoxanide
TNF-α: Tumor necrosis factor-α
PCR: Polymerase chain reaction
